# Multi-targeting NGR-modified liposomes recognizing glioma tumor cells and vasculogenic mimicry for improving anti-glioma therapy

**DOI:** 10.18632/oncotarget.9889

**Published:** 2016-06-07

**Authors:** Dan Huang, Shuang Zhang, Ting Zhong, Wei Ren, Xin Yao, Yang Guo, Xiao-Chuan Duan, Yi-Fan Yin, Shu-Shi Zhang, Xuan Zhang

**Affiliations:** ^1^ Beijing Key Laboratory of Molecular Pharmaceutics and New Drug Delivery Systems, School of Pharmaceutical Sciences, Peking University, Beijing 100191, China; ^2^ Department of Pharmaceutics, School of Pharmaceutical Sciences, Peking University, Beijing 100191, China

**Keywords:** NGR, combretastatin A4, vascular mimicry, glioma, anti-tumor activity

## Abstract

Like the anti-angiogenic strategy, anti-vascular mimicry is considered as a novel targeting strategy for glioma. In the present study, we used NGR as a targeting ligand and prepared NGR-modified liposomes containing combretastatin A4 (NGR-SSL-CA4) in order to evaluate their potential targeting of glioma tumor cells and vasculogenic mimicry (VM) formed by glioma cells as well as their anti-VM activity in mice with glioma tumor cells. NGR-SSL-CA4 was prepared by a thin-film hydration method. The *in vitro* targeting of U87-MG (human glioma tumor cells) by NGR-modified liposomes was evaluated. The *in vivo* targeting activity of NGR-modified liposomes was tested in U87-MG orthotopic tumor-bearing nude mice. The anti-VM activity of NGR-SSL-CA4 was also investigated *in vitro* and *in vivo*. The targeting activity of the NGR-modified liposomes was demonstrated by *in vitro* flow cytometry and *in vivo* biodistribution. The *in vitro* anti-VM activity of NGR-SSL-CA4 was indicated in a series of cell migration and VM channel experiments. NGR-SSL-CA4 produced very marked anti-tumor and anti-VM activity in U87-MG orthotopic tumor-bearing mice *in vivo*. Overall, the NGR-SSL-CA4 has great potential in the multi-targeting therapy of glioma involving U87-MG cells, and the VM formed by U87-MG cells as well as endothelial cells producing anti-U87-MG cells, and anti-VM formed by U87-MG cells as well as anti-endothelial cell activity.

## INTRODUCTION

Glioblastoma is one of the most angiogenic tumors and is characterized by microvascular proliferations [1]. Many anti-angiogenic strategies have been used for the treatment of glioblastoma [2–4]. It has been reported that six different principal cellular mechanisms are associated with tumor angiogenesis, including classical sprouting angiogenesis, vascular co-option, vessel intussusception, vasculogenic mimicry, bone marrow derived vasculogenesis and cancer stem-like cell derived vasculogenesis [4–5]. Vasculogenic mimicry (VM) is defined as a process where tumor cells replace endothelial cells and form a vessel with a lumen [4]. Like tumor angiogenesis, VM is associated with tumor invasion, metastasis and a poor prognosis for cancer patients [6]. The presence of VM in glioblastoma has been confirmed by many reports [7–10]. Like the anti-angiogenic strategy, anti-vascular mimicry is considered as a novel targeting strategy for the treatment of glioma [9].

Ligand-modified drug delivery systems have been used for targeting vascular mimicry [11] and some of them have been successfully used for the treatment of glioma VM [12–13].

Aminopeptidase N (APN) is a membrane-bound, zinc-dependent metalloproteinase that plays a key role in tumor invasion and angiogenesis [14]. A peptide containing the Asn-Gly-Arg (NGR) motif which can recognize a specific isoform of APN has been identified as a potent targeting ligand for the delivery of chemotherapeutic drugs. Our previous research indicated that NGR-modified micelles can target brain microvascular endothelial cells and then produce an anti-glioma effect through anti-angiogenesis [15]. In addition, we have also demonstrated that NGR-modified liposomes can target both HT1080 cells and endothelial cells producing an anti-tumor and anti-angiogenic effect [16]. However, the targeting effect of NGR-modified liposomes on VM is still unclear. Therefore, we speculated that if the NGR could also target VM, it could be used as a targeting VM ligand to produce a drug delivery system with anti-VM activity.

As a vascular disrupting agent, combretastain A4 (CA4) is a tubulin-binding agent that inhibits mitosis and microtubule assembly and is a competitive inhibitor of colchicine binding to tubulin [17–18]. It can induce rapid regression of tumor neovessels through interference with vascular endothelial-cadherin signaling and selective targeting of endothelial cells inducing regression of unstable emerging tumor neovessels by rapidly disrupting molecular engagement of the endothelial cell-specific junctional molecule vascular endothelial-cadherin both *in vitro* and *in vivo*. Anti-brain tumor activity of CA4 has also been reported [19].

Considering the vascular mimicry in glioblastoma, in the present research, we prepared NGR-modified liposomes containing combretastain A4 (NGR-SSL-CA4). The anti-VM and anti-tumor activity of NGR-SSL-CA4 in glioma tumors was investigated *in vitro* and *in vivo*.

## RESULTS

### Preparation and characterization of NGR-SSL-CA4

**Figure 1:**
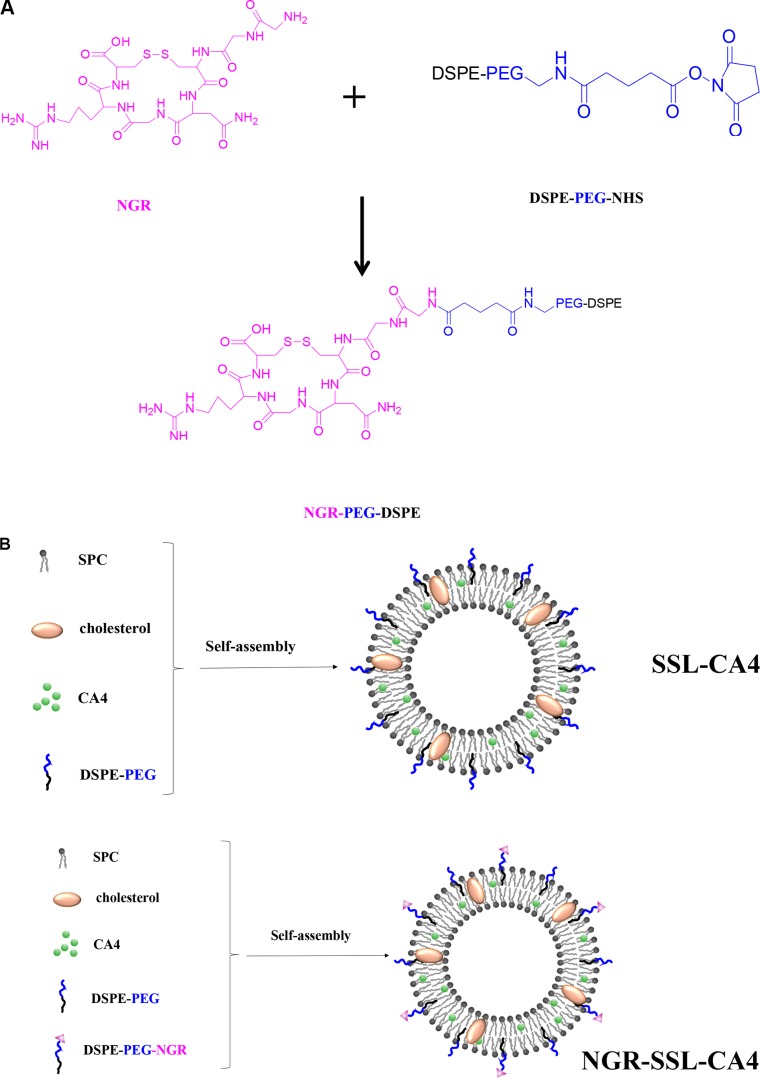

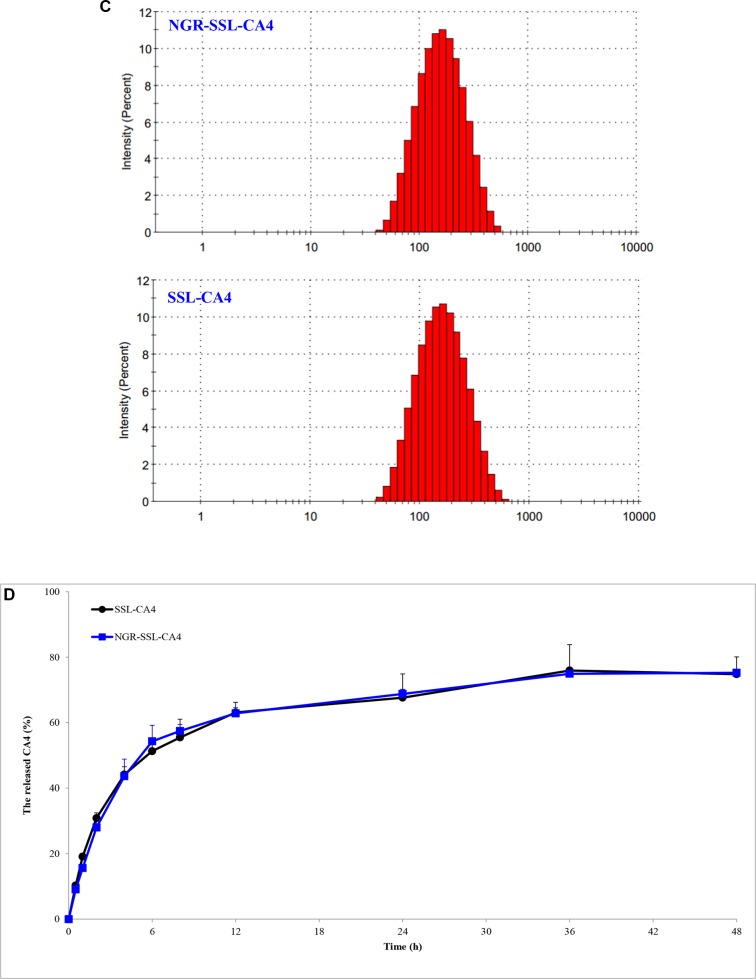
The preparation and characterization of NGR-SSL-CA4 (**A**) Schematic representation of the synthesis of DSPE-PEG-NGR; (**B**) The preparation of SSL-CA4 and NGR-SSL-CA4; (**C**) Typical particle size and distribution of NGR-SSL-CA4 and SSL-CA4; (**D**) The release of CA4 from NGR-SSL-CA4 at 37°C in PBS buffer Data represent the mean ± standard deviation (*n* = 3).

The synthesis of NGR-PEG-DSPE and the preparation of NGR-SSL-CA4 are shown in Figure 1A and 1B.

As shown in Table 1, the average particle size of NGR-SSL-CA4 was about 120 ± 3.33 nm with a polydispersity index (PDI) of 0.287 ± 0.007. A typical particle size and distribution of NGR-SSL-CA4 is shown in Figure 1C. The zeta potential of NGR-SSL-CA4 was negative (−15.6 ± 0.27 mV) (Table 1). The entrapment efficiency of CA4 in NGR-SSL-CA4 was 88.87 ± 3.39% (Table 1). The *in vitro* release results indicated that the release of CA4 from NGR-SSL-CA4 was similar to that of SSL-CA4, demonstrating that the NGR modification did not affect the CA4 release, as shown in Figure 1D. The release of CA4 from free CA4 was also investigated. As shown in Figure S1, the results indicated that the dissolved CA4 could fast transport across the dialysis membrane to the release medium.

**Table 1:** The characterization of NGR-SSL-CA4 (*n* = 3)

Liposomes	Particle size (nm)	Polydensity index	Zeta potential (mV)	Encapsulation efficiency (%)
SSL-CA4	119.7 ± 2.89	0.293 ± 0.002	−22.1 ± 0.21	88.47 ± 7.49
NGR-SSL-CA4	120.0 ± 3.33	0.287 ± 0.007	−15.6 ± 0.27	88.87 ± 3.39

### Flow cytometry analysis

**Figure 2:**
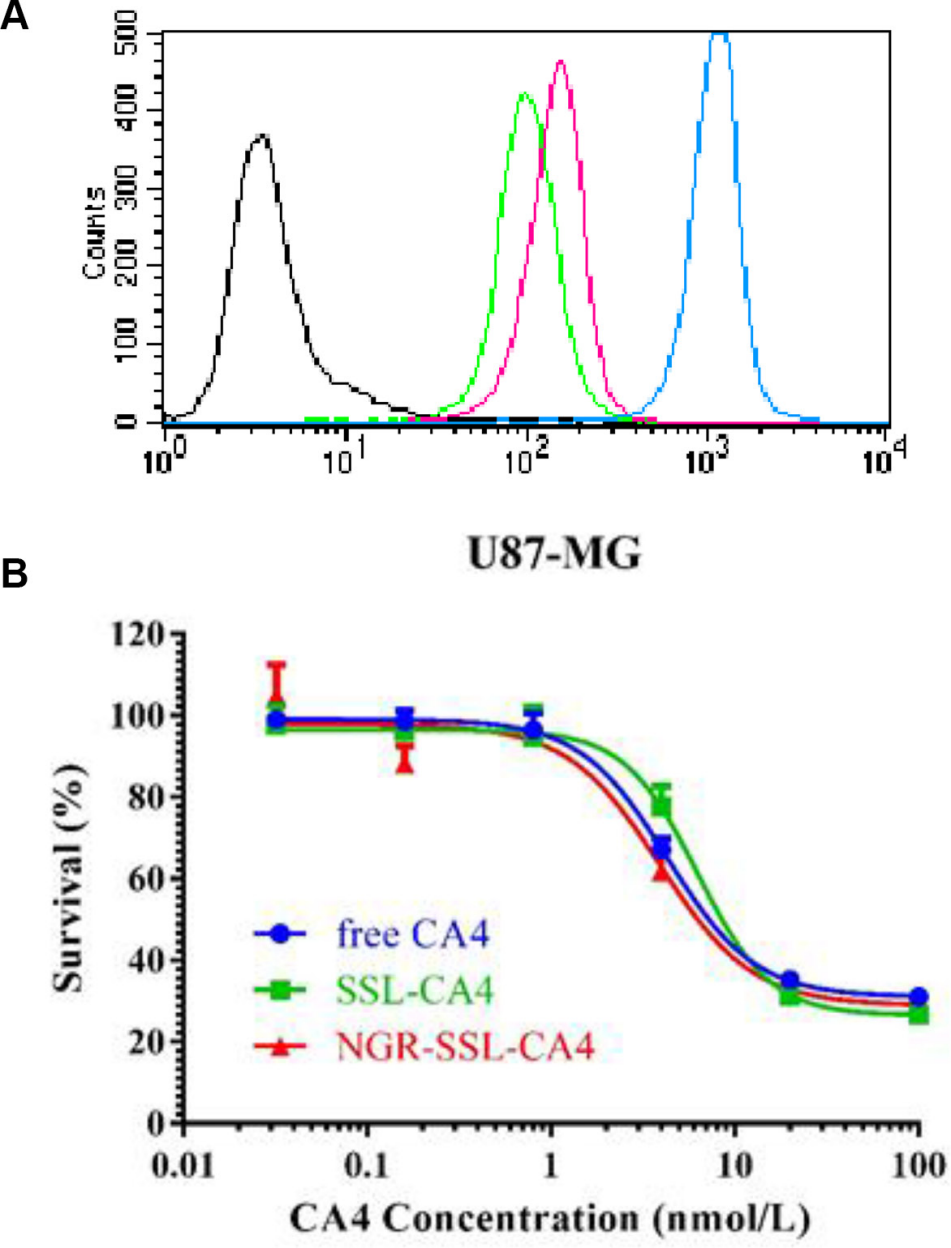
Flow cytometry analysis and the inhibitory effect NGR-SSL-CA4 on U87-MG cells (**A**) The flow cytometric measurement of coumarin-6 uptake from NGR-SSL-coumarin-6 and SSL-coumarin-6 by U87-MG cells at 2 h. Black represents the control, green represents incubation with SSL-coumarin-6, and the peak represents incubation with NGR-SSL-coumarin-6, and brown represents coumarin-6, respectively. (**B**) The inhibitory effect of NGR-SSL-CA4 on U87-MG cell proliferation.

Flow cytometry was used to quantify the total coumarin-6 uptake by U87-MG cells for coumarin-6 formulations. As shown in Figure 2A, the cellular coumarin-6 level for NGR-SSL-coumarin-6 in the U87-MG cells was about 1.4-fold higher than that for SSL-coumarin-6, indicating the targeting effect of NGR-modified liposomes on U87-MG cells.

### Inhibitory effect on U87-MG cell proliferation

The effect of NGR-SSL-CA4 on U87-MG cell proliferation was analyzed by SRB assay as described previously [20]. As shown in Figure 2B, anti-proliferative activity of CA4 in all CA4 formulations was observed in U87-MG cells. The calculated IC_50_ values of CA4 formulations in the U87-MG cell line are shown in Table 2. The results indicated that the *in vitro* anti-tumor activity of NGR-SSL-CA4 in the U87-MG cell line was significantly higher than that of SSL-CA4.

**Table 2:** Cytotoxicity of various CA4 formulations against U87-MG *in vitro* (*n* = 3)

Group	IC_50_ (nM)
free-CA4	4.204 ± 0.705
SSL-CA4	7.795 ± 0.479^##^
NGR-SSL-CA4	4.111 ± 0.286^&&^

## *p* < 0.01 versus free CA4 treatment group;

&& *p* < 0.01 versus SSL-CA4 treatment group.

### Inhibitory effect on U87-MG cell migration

**Figure 3:**
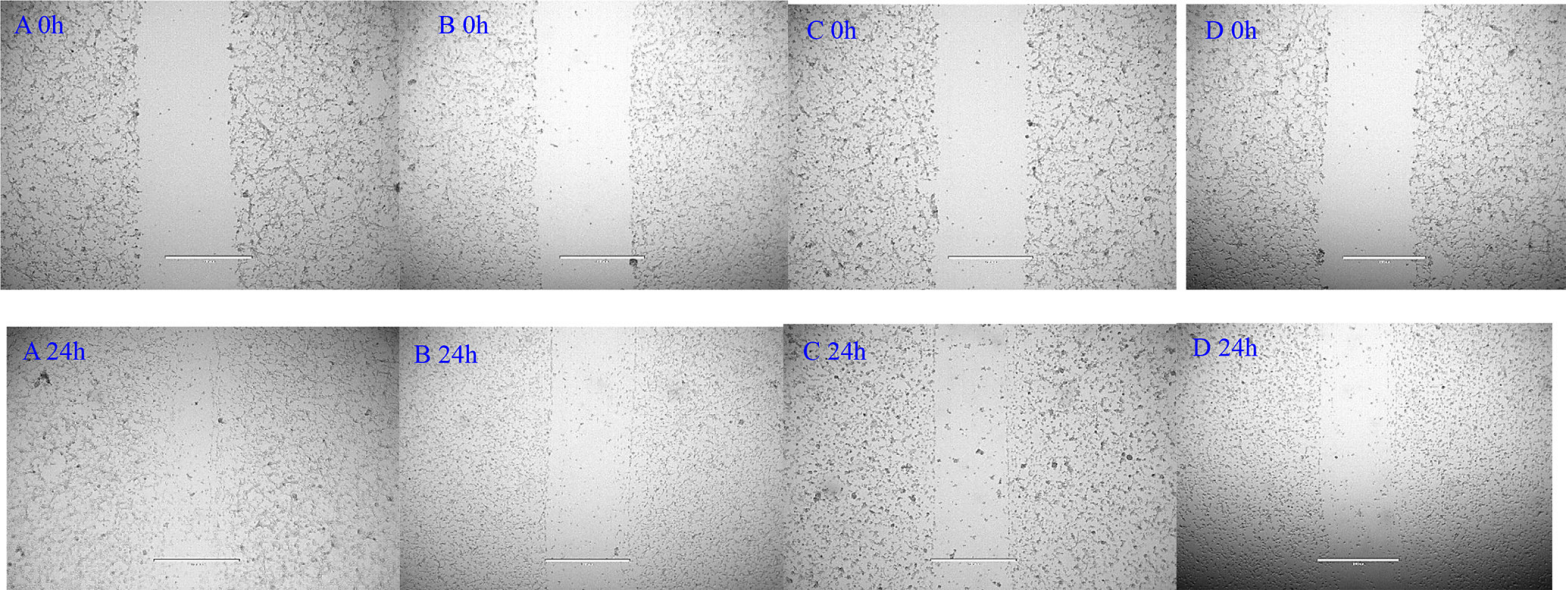
Blocking effect of NGR-SSL-CA4 on wound-healing in U87-MG cells (**A**) control; (**B**) free CA4; (**C**) SSL-CA4; (**D**) NGR-SSL-CA4.

The effect of NGR-SSL-CA4 on U87-MG cell migration was analyzed in the wound assay. For the untreated group, as shown in Figure 3A, the U87-MG cells migrated into the denuded area at 24 h compared with the initial wound scratch at 0 h. In contrast, the migration of U87-MG cells into the denuded areas was significantly inhibited in all CA4 treatment groups (Figure 3B–3D). The anti-migration effect of NGR-SSL-CA4 on U87-MG cells was greater than that of SSL-CA4 (Figure 3D).

### Destructive effect on VM formulation

**Figure 4:**
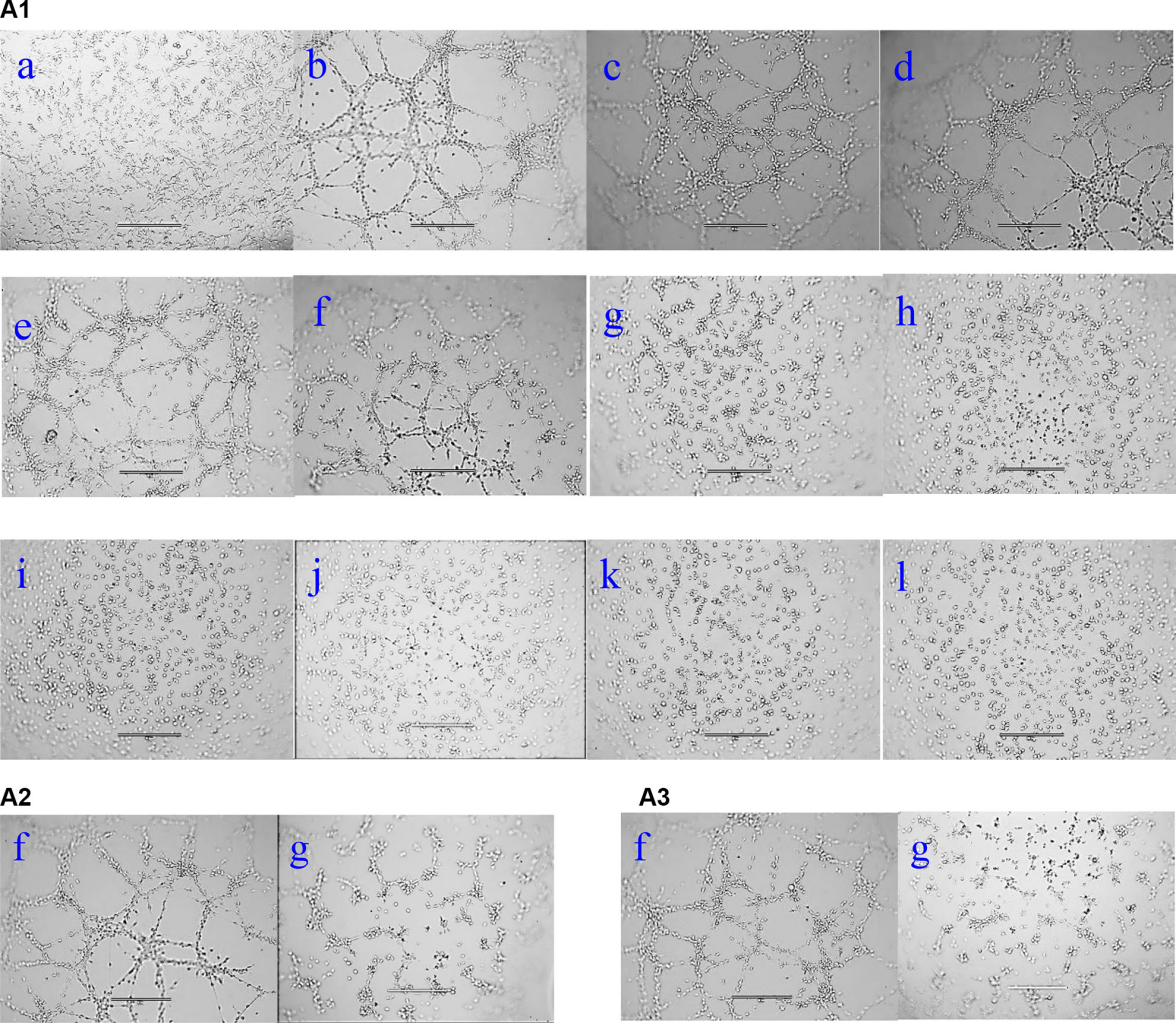
Destructive effect of NGR-SSL-CA4 on U87-MG cell VM channels *in vitro*. (**A1**) free CA4; (**A2**) SSL-CA4; (**A3**) NGR-SSL-CA4. a, cells without matrigel and drug; b, control (0 nM); c, 1 nM; d, 2 nM; e, 4 nM; f, 10 nM; g, 20 nM; h, 40 nM; i, 50 nM; j, 80 nM; k, 100 nM; l, 200 nM. Images were obtained under an EVOSR microscope with a × 10 objective.

A matrigel-based tube formation assay was used to evaluate the formation and destruction of VM. Figure 4 shows the formed VM in the U87-MG cells and the destructive effect of CA4 formulations. The U87-MG cells formed vessel-like loops, channels and networks in matrigel (Figure 4A1 b vs 4A1 a without matrigel). Free CA4 exhibited a marked destructive effect on the VM channels, which was concentration-dependent (Figure 4A1 c – 4A1 l). As shown in Figure 4A1 g, free CA4 exhibited a clear inhibitory effect at concentrations above 20 nM. At a fixed concentration of 10 nM or 20 nM CA4 (Figure 4A2 f and 4A2 g), NGR-SSL-CA4 had a similar destructive effect compared with free CA4 (Figure 4A1 f and 4A1 g) and a greater destructive effect than SSL-CA4 (Figure 4A3 f and 4A3 g).

### Regulating protein indicators of the VM channels

**Figure 5:**
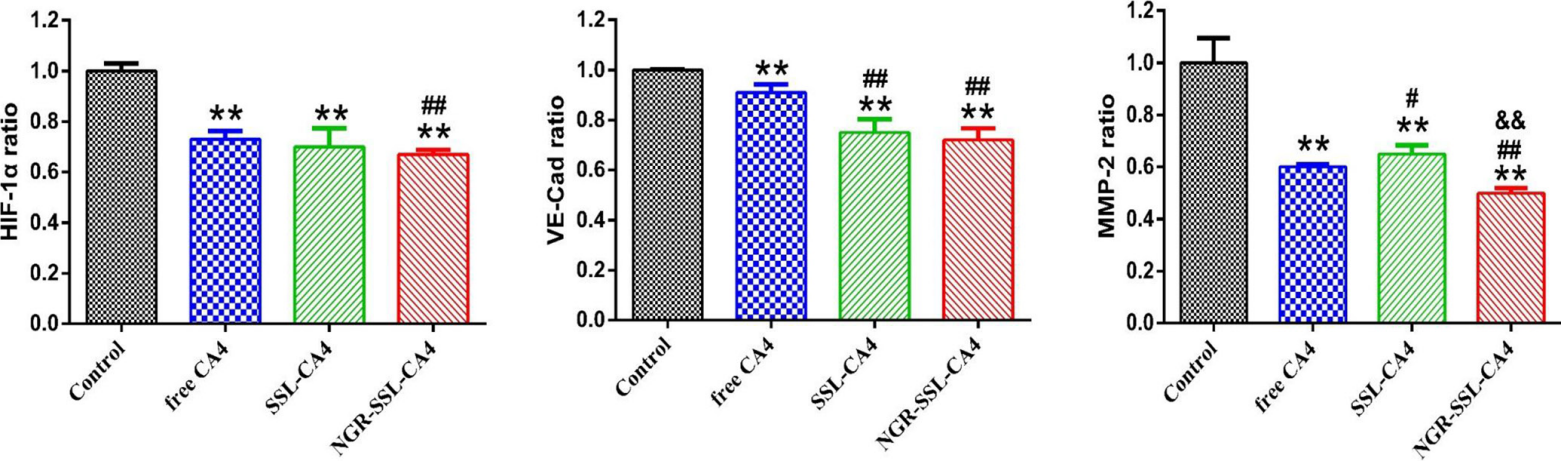
The regulatory effect of NGR-SSL-CA4 on HIF-1α, VE-Cad and MMP-2 VM protein indicators Data are the mean ± standard deviation (*n* = 3). **p < 0.01 versus control group; ^#^*p* < 0.05 or ^##^*p* < 0.01 versus free CA4 treatment group; ^&&^*p* < 0.01 versus SSL-CA4 treatment group.

Three protein indicators, hypoxia inducible factor- 1α (HIF-1α), VE-Cad and MMP-2, which could be involved in the formation of VM channels, were selected to investigate the mechanism of action of NGR-SSL- CA4 on VM. Figure 5 shows the regulatory effects on the indicators of the VM channels in U87-MG cells after treatment with CA4 formulations. The results obtained showed that all protein indicators, including HIF-1α, VE-Cad and MMP-2, were clearly down-regulated after treatment with CA4 formulations. Compared with free CA4, NGR-SSL-CA4 significantly reduced the activity of HIF-1α, VE-Cad and MMP-2 in U87-MG cells. The effect of NGR-SSL-CA4 on down-regulated HIF-1α and VE-Cad was a little greater than that of SSL-CA4, and significantly greater than that of SSL-CA4 in MMP-2.

### *In vivo* imaging

**Figure 6:**
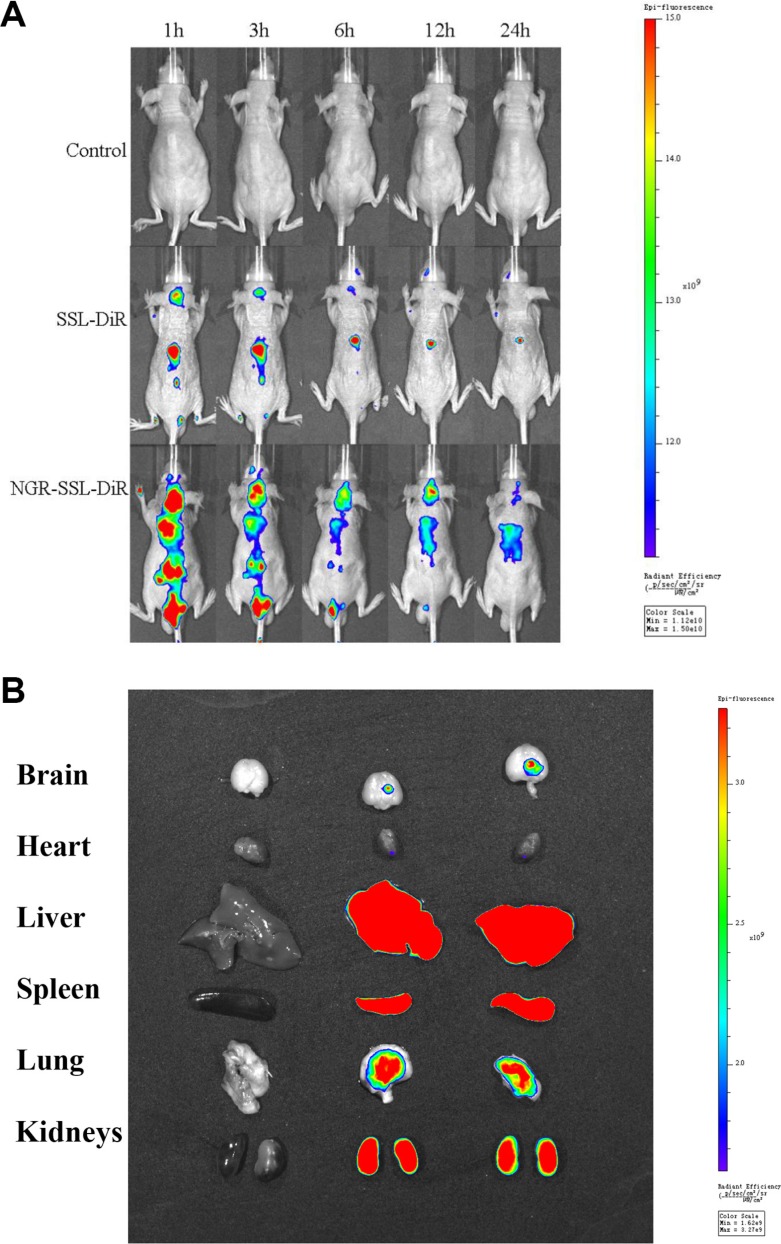
*In vivo* real-time imaging The U87-MG orthotopic tumor-bearing nude mice were given a tail vein injection of 5% glucose injection, SSL-DiR and NGR-SSL-DiR. All mice were scanned at 1, 3, 6, 12 and 24 hours (**A**). The mice were sacrificed at 24 h and the brain, heart, liver, spleen, lung, and kidneys were collected immediately. The fluorescence signal intensities in different tissues were scanned (**B**).

Figure 6 shows the distribution and tumor accumulation of fluorescent DiR in U87-MG orthotopic tumor-bearing nude mice. The results indicated that NGR-SSL-DiR has a stronger fluorescence signal in the brain than SSL-DiR at all observed time points (Figure 6A). The major organs (heart, liver, spleen, lung, kidneys) and tumor-bearing brain tissues were excised for *ex vivo* examination at 24 h post- injection. The results obtained showed that a higher fluorescence intensity was found in tumor tissue after administration of NGR-SSL-DiR compared with SSL-DiR (Figure 6B).

### *In vivo* anticancer activity

**Figure 7:**
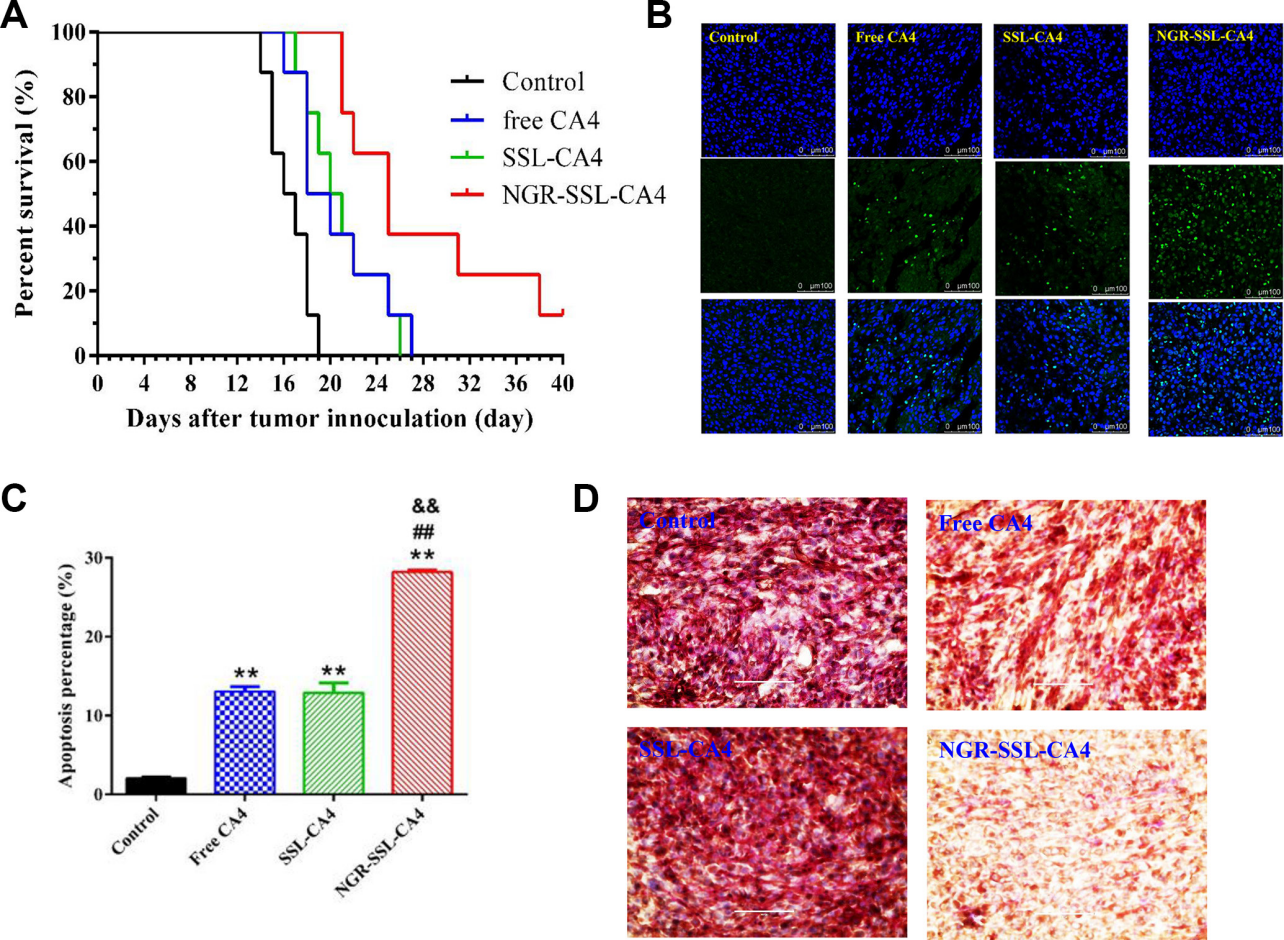
*In vivo* anti-tumor activity of NGR-SSL-CA4 in U87-MG orthotopic glioma tumor-bearing nude mice (**A**) Kaplan–Meier survival curves. Kaplan–Meier survival curves of U87-MG orthotopic glioma tumor-bearing nude mice treated with 5% glucose injection (black), free CA4 (blue), SSL-CA4 (green) and NGR-SSL-CA4 (red). The CA4 formulations were all given by tail vein injection on days 8, 10, 12 and 14, at a dose of 15 mg/kg. The total dose of CA4 in all treatment groups was 60 mg/kg. The results indicate that the median survival time of U87-MG tumor-bearing nude mice treated with NGR-SSL-CA4 (25 days) was significantly longer than that of mice treated with a 5% glucose injection (16.5 days, *p* < 0.01), SSL-CA4 (20.5 days, *p* < 0.01) and free CA4 (19.0 days, *p* < 0.01). (**B**) TUNEL staining of the tumor tissues was performed according to the standard protocols provided by the manufacturers. (**C**) The fluorescence area of each group was used for the statistical analysis of apoptosis activity. (**D**) CD3-PAS dual staining (final magnification, ×10). **p < 0.01 versus control group; ^##^*p* < 0.01 versus free CA4 treatment group; ^&&^*p* < 0.01 versus SSL-CA4 treatment group.

The anti-tumor effect of NGR-SSL-CA4 was evaluated in U87-MG orthotopic tumor-bearing nude mice. The Kaplane-Meier survival curves showed that the median survival time of U87-MG tumor-bearing nude mice treated with NGR-SSL-CA4 (25 days) was significantly longer than that of mice treated with 5 % glucose injection (16.5 days, *p* < 0.01), SSL-CA4 (20.5 days, *p* < 0.01) and free CA4 (19.0 days, *p* < 0.01), as shown in Figure 7A.

The effect of tumor cell apoptosis was investigated by TUNEL analysis staining of tumor tissue sections. As shown in Figure 7B, tumors from the NGR-SSL-CA4-treated group exhibited more advanced cell apoptosis compared with the groups treated with 5% glucose injection, free CA4 and SSL-CA4. The calculated results are shown in Figure 7C.

To evaluate the anti-VM activity of NGR-SSL-CA4, the VM density was assessed by CD34-PAS dual staining. As shown in Figure 7D, fewer VM channels were observed in the NGR-SSL-CA4 treatment group, indicating the destruction of VM channels. Also, the SSL-CA4 treatment group exhibited only a negligible destruction of VM channels, which was similar to the results in the free CA4 treatment group.

## DISCUSSION

It has been reported that traditional anti-angiogenic therapy in glioma fails to show an overall survival benefit [21]. We believed that one possible reason might be that the drugs used in traditional anti-angiogenic therapy tend to be anti-VEGF agents which play a role mainly by reducing endothelial cell proliferation or inducing endothelial cell apoptosis, while these drugs have little effect on vasculogenic mimicry (VM) formed by tumor cells. Many chemotherapeutic agents, including paclitaxel or plus artemether, epirubicin plus celecoxib, vincristine plus dasatinib, ginsenoside Rg3, endostatin, all-trans retinoic acid and dehydroeffusol, have been used for anti-VM [13, 22–27].

CA4 is a vascular disrupting agent. In the present research, CA4 was selected as the model drug. The *in vitro* results of the inhibitory effect on tumor cells, the inhibitory effect on cell migration and the destructive effect on VM channels proved that CA4 has an anti-MV effect in U87-MG cells. It has been reported that the main VM signaling pathways involve: up-regulation of VE-Cad by HIF-1α mediating the phosphorylation of EphA2, followed by a cascade of cellular phosphorylated responses, resulting in remodeling the extracellular matrix by MMPs [28–29]. Our results indicated that CA4 could down-regulate the expressions of HIF-1α, VE-Cad and MMP-2 protein indicators in U87-MG cells, producing an inhibitory effect on VM channels. All above results confirm that CA4 exhibits an inhibitory effect on U87-MG cells proliferation, migration and formation of VM.

Some ligand-modified drug-loaded nanocarriers have also been used for the treatment of VM, such as cell-penetrating peptide R8 and PTD_HIV-1_, ligand targeting to BBB like MAN and cRGD, targeting to receptors which are highly expressed in tumor endothelial vessels and(or) VM channels like K237 and VEGF-PE38, peptides targeting to cancer cells or cancer stem cells like DQA and CVNHPAFAC-NH2. In addition, some ligand-modified drug-loaded delivery systems have been successfully prepared for the treatment of VM in glioma [12–13, 22–23, 30].

In the present research, NGR-SSL-CA4 was prepared and its anti-VM effect was evaluated *in vitro* and *in vivo*. The targeting effect of NGR-modified liposomes on U87-MG cells was confirmed in our flow cytometry experiments. The inhibitory effect on cell migration and the destructive effect of NGR-SSL-CA4 on VM channels were greater than that of SSL-CA4 in U87- MG cells, indicating the targeting role of NGR on VM. Similar results were also observed in down-regulating the expressions of HIF-1α, VE-Cad and MMP-2 protein in the experiments involving U87-MG cells.

The anti-tumor activity of NGR-SSL-CA4 was higher than that of SSL-CA4. Considering the results of the tumor cell apoptosis experiments, the *in vitro* destructive effect on the VM channels and *in vivo* CD34- PAS dual staining experiments, and the anti-angiogenic effect in the reported reference, we suggest that NGR-SSL-CA4 can target U87-MG cells, and the VM formed by U87-MG cells as well as endothelial cells compared with SSL- CA4. In view of the *in vitro* anti-tumor activity, the anti-VM effect and the reported vascular disruptive effect of CA4, we suggest that NGR-SSL-CA4 is more effective in killing U87-MG cells, the VM formed by U87- MG cells as well as endothelial cells than SSL-CA4.

The strategy of multi-targeting peptide-modified nanoparticles for recognizing vasculogenic mimicry, tumor neovasculature and glioma cells has been reported [13]. This CK peptide is composed of a human sonic hedgehog (SHH) targeting peptide (CVNHPAFAC) and a KDR targeting peptide (K237 HTMYYHHYQHHL) through a GYG linker. Compared with this 24 amino acid CK, our cyclic NGR peptide (GGCNGRC) is a pentapeptide which is able to target U87-MG cells as well as the VM formed by U87-MG cells. In addition, NGR is a targeting endothelial cell ligand. Therefore, this simple cyclic NGR peptide has similar multi-targeting activity to CK peptide, indicating its important role on targeting U87-MG cells, VM formed by U87-MG cells as well as endothelial cells.

## MATERIALS AND METHODS

### Materials and animals

Combretastatin A4 (CA4) was purchased from Shanghai Fude Chemicals, Co. Ltd. (Shanghai, China). Soybean phosphatidylcholine (SPC) was provided by Lipoid GmbH (Ludwigshafen, Germany). DSPE-PEG_2000_ (DSPE-PEG) and DSPE-PEG_2000_-NHS (DSPE-PEG-NHS) were obtained from NOF Corporation (Tokyo, Japan). The cyclic NGR peptide, GGCNGRC, was synthesized by Beijing Scilight Biotechnology Co., Ltd. (Beijing, China). NGR-PEG-DSPE was synthesized according to our previously reported method [15–16]. Near-infrared lipophilic carbocyanine dye 1,10-dioctadecyltetramethyl indotricarbocyanine iodide (DiR), coumarin-6, cholesterol and sulforhodamine B were purchased from Sigma-Aldrich (St Louis, MO, USA). 4′,6-Diamidino-2-phenylindole (DAPI) solution was obtained from Beyotime Institute of Biotechnology (Shanghai, China).

Cell culture medium, MEM medium, was obtained from Macgene Biotech Co. Ltd. (Beijing China). Penicillin-streptomycin and fetal bovine serum (FBS) were obtained from GIBCO (Invitrogen Co. (Carlsbad, USA)). All other reagents were of analytical or HPLC grade.

Human glioma U87-MG cells were purchased from the Cell Resource Center, Peking Union Medical College (Beijing, China). The cancer cells were cultured at 37°C under an atmosphere of 5% CO2. The culture medium was MEM medium supplemented with 10% FBS and 1% nonessential amino acid solution.

Male BALB/C nude mice weighing 19–21 g (7–8 weeks) were obtained from the Experimental Animal Center of Peking University Health Science Center. All care and handling of the animals were performed with the approval of Institutional Authority for Laboratory Animal Care of Peking University.

### Preparation of NGR-SSL-CA4

The NGR-modified liposomes containing CA4 (NGR-SSL-CA4) were prepared by the thin-film hydration method, as described previously [16]. Briefly, a mixture of CA4, SPC, cholesterol, PEG-DSPE and NGR-PEG-DSPE (The molar ratio of NGR-PEG-DSPE:PEG-DSPE:CHOL:SPC was 5:5:25:65, the weight ratio of lipid:CA4 was 20:1. The modification degree of NGR in NGR-SSL-CA4 was about 5% (molar ratio %)) was dissolved in chloroform. Then, the solvent was evaporated using an RE52 rotary evaporator (Shanghai Yarong Biochemistry Instrument Company, China) in a round-bottomed flask at 40°C for about 40 minutes to obtain a solid film. This film was then flushed with nitrogen gas for 30 minutes and stored overnight in a desiccator to remove any traces of chloroform. After that, the thin-film was hydrated in a 5 % glucose solution by sonication in a water bath for 10 min to produce a suspension of liposomes. The liposome suspension was extruded 10 times through a polycarbonate membrane (Millipore, Bedford, MA, USA) with a pore size of 100 nm. The free CA4 was then separated on a Sephadex G-50 column by eluting with 5% glucose solution.

For the preparation of sterically stabilized liposomes containing CA4 (SSL-CA4), a similar procedure was carried out except that the NGR-PEG-DSPE was replaced by PEG-DSPE.

The preparation of liposomes loaded with coumarin-6 or DiR (NGR-SSL-coumarin-6, SSL-coumarin-6, NGR-SSL-DiR and SSL-DiR), was carried out by a similar procedure except that the CA4 was replaced by coumarin-6 or DiR.

### Characterization of NGR-SSL-CA4

The particle size and zeta potential of NGR-SSL-CA4 were measured using a Malvern Zeta Sizer Nano-ZS (Malvern, UK) at 25°C. The encapsulation efficiency was estimated from the following formula:

$$\text{Encapsulation Efficiency}=\frac{\text{Actual amount of drug loaded in liposomes}}{\text{Theoretical amount of drug loaded in liposomes}}\times 100\%$$

### *In vitro* release of CA4 from NGR-SSL-CA4

The release of CA4 from NGR-SSL-CA4 was investigated by dialysis. Briefly, a sample of NGR-SSL-CA4 (0.1 ml, 1 mg/ml) was placed in a dialysis tube (MWCO 7000) and tightly sealed. Then, the dialysis tube was immersed in 40 ml release medium (PBS 7.4) and incubated in an orbital shaker at 37°C. Samples (0.2 ml) were removed at predetermined time intervals from the release medium over a period of 48 h, and these were replaced by a similar volume of fresh medium. The concentration of CA4 was determined by HPLC.

### Flow cytometry

U87-MG cells were seeded at a density of 4 × 10^5^ cells/well in 12-well plates and incubated at 37°C for 24 h to allow cell attachment. After 24 h, the medium was replaced with coumarin-6 solution or different coumarin-6-loaded liposomes (the concentration of coumarin-6 was 300 ng/ml). After 2 h incubation, the cells were washed 3 times with PBS. Then, the cells were harvested by trypsinization and centrifuged at 1000 rpm for 5 min and resuspended in 500 μl PBS and examined by flow cytometry using an FACScan instrument (Becton Dickinson, San Jose, CA, USA). Cell-associated coumarin-6 was excited with an argon laser (467 nm), and fluorescence was detected at 502 nm.

### *In vitro* cytotoxicity of NGR-SSL-CA4

The cytotoxicity of NGR-SSL-CA4 against U87- MG cells was measured by sulforhodamine B (SRB) assay [31]. Briefly, U87-MG cells were seeded at a density of 8000 cells/well in 96-well culture plates, and cultured for 24 h. After cell adhesion, NGR-SSL-CA4 was added to each well in complete medium. After incubation for another 48 h at 37°C, cells were fixed with trichloroacetic acid, washed and stained with SRB. After shaking, the absorbance at 540 nm was recorded using a 96-well plate reader (Bio-tek, 808, USA). The survival rates were calculated using the following formula: Survival % = (A_540__nm_ for treated cells/A_540__nm_ for control cells) × 100%, where A_540__nm_ represents the absorbance. Each assay was repeated a minimum of three times with quadruplicate determinations per dose level.

### Blocking the wound-healing *in vitro*

The blocking effect of NGR-SSL-CA4 on wound-healing was measured using U87-MG cells. Briefly, confluent U87-MG cells in 12-well plates (Costar, America) were mechanically “wounded” by scraping away a swath of cells with a pipette tip and denuding a strip of the monolayer 300 μm wide [32]. The cells were then washed with PBS, followed by addition of free CA4, SSL-CA4 and NGR-SSL-CA4 at a concentration of 10 nM CA4 (diluted with serum-free medium). Serum-free medium without drug was used as a control. After a 24 h incubation, images of the scratch wounds were photographed at 0 h and 24 h using an EVOS microscope with the same magnification.

### Destruction of cancer VM channels *in vitro*

The destroying effect of NGR-SSL-CA4 on the VM channels was examined on U87-MG cells using a three-dimensional (3D) culture model [33]. Briefly, a 96-well culture plate was coated with matrigel (50 μl/well, BD Biosciences, USA) at 37°C for 30 min. U87-MG cells were seeded at 1 × 10^4^. Then free CA4 (1.0-200 nM), SSL-CA4 (10 nM and 20 nM) and NGR-SSL-CA4 (10 nM and 20 nM) were added to the culture system. Free CA4 was prepared by dissolving in 1% DMSO and then all formulations were diluted with serum-free medium. After treatment with CA4 formulations, the cells were incubated for 12 h, and then photographed and analyzed using an EVOS microscope (AMG, USA). Cells incubated without matrigel and CA4 were included as a negative control while cells incubated with matrigel and CA4 free were used as a positive control.

### Regulation of cancer VM indicators

Briefly, U87-MG cells were cultured for 24 h, and then treated with free CA4, SSL-CA4 and NGR-SSL-CA4 at a concentration of 50 nM CA4 (diluted with serum-free medium). After incubation for 12 h, the cells were harvested and lysed. The cell lysates were analyzed using a microplate reader according to the manufacturer's instructions supplied with the enzyme-linked immunosorbent assay kits (Cusabio Biotech Co. Ltd., Beijing, China) for HIF-1α, VE-Cad and MMP-2.

### *In vivo* imaging in mice

The U87-MG orthotopic glioma model was established by injection of U87-MG cells (5 × 10^5^ cells/3 μl of PBS (pH7.4)) into the right brain of each nude mouse (2 mm laterally to the bregma and 3.0 mm deep from the dura) using a stereotaxic apparatus [34]. On the 10th day, the mice were given a tail vein injection of 5% glucose injection, SSL-DiR and NGR-SSL-DiR. *In vivo* fluorescence imaging was performed using an IVIS SPECTRUM *in vivo* imaging system (Xenogen, USA) at different times (1, 3, 6, 12 and 24 h). The mice were sacrificed at 24 h and brain, heart, liver, spleen, lung, and kidneys were collected immediately. The fluorescence intensities in the different tissues were photographically recorded.

### *In vivo* anti-tumor activity

The U87-MG orthotopic glioma model was established as described above. On the 8th day, the mice were randomly assigned to four groups (12 animals per group): group 1 was given a 5% glucose injection, group 2 was given free CA4, group 3 was given SSL-CA4 and group 4 was given NGR-SSL-CA4. The CA4 formulations were all given via the tail vein on days 8, 10, 12 and 14, at a dose of 15 mg/kg. The total dose of CA4 in all treatment groups was 60 mg/kg. Throughout the study, mice were weighed regularly. On day 16 after tumor inoculation, one or two mice in each group were executed and the brain was collected for the preparation of histological sections. TUNEL staining of the tissue sections was performed according to the standard protocols provided by the manufacturers. CD34 and periodic acid-Schiff (PAS) dual staining was used to measure the destructive effect on VM channels in the tumor tissues by immunohistochemistry according to the standard protocols provided by the manufacturers. The survival time was calculated from the day of U87-MG cell inoculation (0 day) to the day of death. Kaplan-Meier survival curves were plotted for each group.

### HPLC analysis of CA4

The concentration of CA4 in NGR-SSL-CA4 was determined by HPLC using a Waters HPLC system consisting of a 1525-pump, and a 2487-ultraviolet detector (Waters Co. Inc., Westerville, OH, USA). The mobile phase, consisting of methanol-water (68:32, v/v), was delivered at a flow rate of 1 ml/min. Chromatographic separation was performed on a Phenomenex ODS3 column (250 × 4.6 mm, 5 mm, Torrance, CA, USA) and the detector wavelength was 295 nm.

### Statistical analysis

All data are presented as the mean ± standard deviation (SD). One-way analysis of variance (ANOVA) was used to determine significance among groups, after which post-hoc tests with the Bonferroni correction were used for comparison between individual groups. Statistical significance was established at *p* < 0.05.

## CONCLUSIONS

In summary, we prepared NGR-SSL-CA4 with the aim of evaluating its potential targeting of glioma U87-MG cells as well as VM formed by U87-MG cells and examined its anti-tumor and anti-MV activity. The targeting activity of the NGR-modified liposomes was demonstrated by *in vitro* flow cytometry as well as cell migration and the destructive effect in VM channel experiments. The results of the *in vitro* cell migration and destruction of VM channels as well as *in vivo* cell apoptosis and CD34-PAS dual staining confirmed the anti-tumor and anti-VM activity of NGR-SSL-CA4 *in vitro* and *in vivo*. Overall, the NGR-SSL-CA4 prepared in the present research has great potential in the multi-targeting therapy of glioma targeting the U87-MG cells, the VM formed by U87-MG cells as well as endothelial cells producing anti-U87-MG cells, anti-VM formed by U87-MG cells as well as anti-endothelial cell activity.

## SUPPLEMENTARY MATERIALS



## References

[1] Ricci-Vitiani L, Pallini R, Biffoni M, Todaro M, Invernici G, Cenci T, Maira G, Parati EA, Stassi G, Larocca LM, De Maria R (2010). Tumour vascularization via endothelial differentiation of glioblastoma stem-like cells. Nature.

[2] Arrillaga-Romany I, Reardon DA, Wen PY (2014). Current status of antiangiogenic therapies for glioblastomas. Expert Opin Investig Drugs.

[3] De Bonis P, Marziali G, Vigo V, Peraio S, Pompucci A, Anile C, Mangiola A (2013). Antiangiogenic therapy for high-grade gliomas: current concepts and limitations. Expert Rev Neurother.

[4] Plate KH, Scholz A, Dumont DJ (2012). Tumor angiogenesis and anti-angiogenic therapy in malignant gliomas revisited. Acta Neuropathol.

[5] Jain RK, Carmeliet P (2012). SnapShot: Tumor angiogenesis. Cell.

[6] Qiao L, Liang N, Zhang J, Xie J, Liu F, Xu D, Yu X, Tian Y (2015). Advanced research on vasculogenic mimicry in cancer. J Cell Mol Med.

[7] Wang SY, Ke YQ, Lu GH, Song ZH, Yu L, Xiao S, Sun XL, Jiang XD, Yang ZL, Hu CC (2013). Vasculogenic mimicry is a prognostic factor for postoperative survival in patients with glioblastoma. J Neurooncol.

[8] Huang M, Ke Y, Sun X, Yu L, Yang Z, Zhang Y, Du M, Wang J, Liu X, Huang S (2014). Mammalian target of rapamycin signaling is involved in the vasculogenic mimicry of glioma via hypoxia-inducible factor-1α. Oncol Rep.

[9] Chen YS, Chen ZP (2014). Vasculogenic mimicry: a novel target for glioma therapy. Chin J Cancer.

[10] Scully S, Francescone R, Faibish M, Bentley B, Taylor SL, Oh D, Schapiro R, Moral L, Yan W, Shao R (2012). Transdifferentiation of glioblastoma stem-like cells into mural cells drives vasculogenic mimicry in glioblastomas. J Neurosci.

[11] Ying M, Chen G, Lu W (2015). Recent Advances and Strategies in Tumor Vasculature Targeted Nano-Drug Delivery Systems. Curr Pharm Des.

[12] Liu Y, Mei L, Yu Q, Xu C, Qiu Y, Yang Y, Shi K, Zhang Q, Gao H, Zhang Z, He Q (2015). Multifunctional Tandem Peptide Modified Paclitaxel-Loaded Liposomes for the Treatment of Vasculogenic Mimicry and Cancer Stem Cells in Malignant Glioma. ACS Appl Mater Interfaces.

[13] Feng X, Yao J, Gao X, Jing Y, Kang T, Jiang D, Jiang T, Feng J, Zhu Q, Jiang X, Chen J (2015). Multi-targeting Peptide-Functionalized Nanoparticles Recognized Vasculogenic Mimicry, Tumor Neovasculature, and Glioma Cells for Enhanced Anti-glioma Therapy. ACS Appl Mater Interfaces.

[14] Arap W, Pasqualini R, Ruoslahti E (1998). Cancer treatment by targeted drug delivery to tumor vasculature in a mouse model. Science.

[15] Zhao BJ, Ke XY, Huang Y, Chen XM, Zhao X, Zhao BX, Lu WL, Lou JN, Zhang X, Zhang Q (2011). The antiangiogenic efficacy of NGR-modified PEG-DSPE micelles containing paclitaxel (NGR-M-PTX) for the treatment of glioma in rats. J Drug Target.

[16] Luo LM, Huang Y, Zhao BX, Zhao X, Duan Y, Du R, Yu KF, Song P, Zhao Y, Zhang X, Zhang Q (2013). Anti-tumor and anti-angiogenic effect of metronomic cyclic NGR-modified liposomes containing paclitaxel. Biomaterials.

[17] Nagaiah G, Remick SC (2010). Combretastatin A4 phosphate: a novel vascular disrupting agent. Future Oncol.

[18] Gridelli C, Rossi A, Maione P, Rossi E, Castaldo V, Sacco PC, Colantuoni G (2009). Vascular disrupting agents: a novel mechanism of action in the battle against non-small cell lung cancer. Oncologist.

[19] Liu L, Mason RP, Gimi B (2015). Dynamic bioluminescence and fluorescence imaging of the effects of the antivascular agent Combretastatin-A4P (CA4P) on brain tumor xenografts. Cancer Lett.

[20] Huang Y, Chen XM, Zhao BX, Ke XY, Zhao BJ, Zhao X, Wang Y, Zhang X, Zhang Q (2010). Antiangiogenic Activity of Sterically Stabilized Liposomes Containing Paclitaxel (SSL-PTX): *In Vitro* and *In Vivo*. AAPS PharmSciTech.

[21] Batchelor TT, Reardon DA, de Groot JF, Wick W, Weller M (2014). Antiangiogenic therapy for glioblastoma: current status and future prospects. Clin Cancer Res.

[22] Ju RJ, Li XT, Shi JF, Li XY, Sun MG, Zeng F, Zhou J, Liu L, Zhang CX, Zhao WY, Lu WL (2014). Liposomes, modified with PTD(HIV-1) peptide, containing epirubicin and celecoxib, to target vasculogenic mimicry channels in invasive breast cancer. Biomaterials.

[23] Li XY, Zhao Y, Sun MG, Shi JF, Ju RJ, Zhang CX, Li XT, Zhao WY, Mu LM, Zeng F, Lou JN, Lu WL (2014). Multifunctional liposomes loaded with paclitaxel and artemether for treatment of invasive brain glioma. Biomaterials.

[24] Guo JQ, Zheng QH, Chen H, Chen L, Xu JB, Chen MY, Lu D, Wang ZH, Tong HF, Lin S (2014). Ginsenoside Rg3 inhibition of vasculogenic mimicry in pancreatic cancer through downregulation of VE-cadherin/EphA2/MMP9/MMP2 expression. Int J Oncol.

[25] Chen X, Zhang H, Zhu H, Yang X, Yang Y, Yang Y, Min H, Chen G, Liu J, Lu J, Cheng H, Sun X (2016). Endostatin combined with radiotherapy suppresses vasculogenic mimicry formation through inhibition of epithelial-mesenchymal transition in esophageal cancer. Tumour Biol.

[26] Ling GQ, Liu YJ, Ke YQ, Chen L, Jiang XD, Jiang CL, Ye W (2015). All-trans retinoic acid impairs the vasculogenic mimicry formation ability of U87 stem-like cells through promoting differentiation. Mol Med Rep.

[27] Liu W, Meng M, Zhang B, Du L, Pan Y, Yang P, Gu Z, Zhou Q, Cao Z (2015). Dehydroeffusol effectively inhibits human gastric cancer cell-mediated vasculogenic mimicry with low toxicity. Toxicol Appl Pharmacol.

[28] Qiao L, Liang N, Zhang J, Xie J, Liu F, Xu D, Yu X, Tian Y (2015). Advanced research on vasculogenic mimicry in cancer. J Cell Mol Med.

[29] Paulis YW, Soetekouw PM, Verheul HM, Tjan-Heijnen VC, Griffioen AW (2010). Signalling pathways in vasculogenic mimicry. Biochim Biophys Acta.

[30] Zhang Y, Sun X, Huang M, Ke Y, Wang J, Liu X (2015). A novel bispecific immunotoxin delivered by human bone marrow-derived mesenchymal stem cells to target blood vessels and vasculogenic mimicry of malignant gliomas. Drug Des Devel Ther.

[31] Vichai V, Kirtikara K (2006). Sulforhodamine B colorimetric assay for cytotoxicity screening. Nat Protoc.

[32] Bijman MN, van Nieuw Amerongen GP, Laurens N, van Hinsbergh VW, Boven E (2006). Microtubule-targeting agents inhibit angiogenesis at subtoxic concentrations, a process associated with inhibition of Rac1 and Cdc42 activity and changes in the endothelial cytoskeleton. Mol Cancer Ther.

[33] Cui YF, Liu AH, An DZ, Sun RB, Shi Y, Shi YX, Shi M, Zhang Q, Wang LL, Feng Q, Pan GL, Wang Q (2015). Claudin-4 is required for vasculogenic mimicry formation in human breast cancer cells. Oncotarget.

[34] Zhao Y, Ren W, Zhong T, Zhang S, Huang D, Guo Y, Yao X, Wang C, Zhang WQ, Zhang X, Zhang Q (2016). Tumor-specific pH-responsive peptide-modified pH-sensitive liposomes containing doxorubicin for enhancing glioma targeting and anti-tumor activity. J Control Release.

