# Supplementary material for: Multi-targeting NGR-modified liposomes recognizing glioma tumor cells and vasculogenic mimicry for improving anti-glioma therapy
Source: Oncotarget. 2016 Jun 7;7(28):43616–28. doi: 10.18632/oncotarget.9889 (PMC5190048; doi:10.18632/oncotarget.9889)
Supplement: Supplementary file 1 [file oncotarget-07-43616-s001.pdf]

Multi-targeting NGR-modified liposomes recognizing glioma tumor cells and vasculogenic mimicry for improving anti-glioma therapy

Supplementary Materials

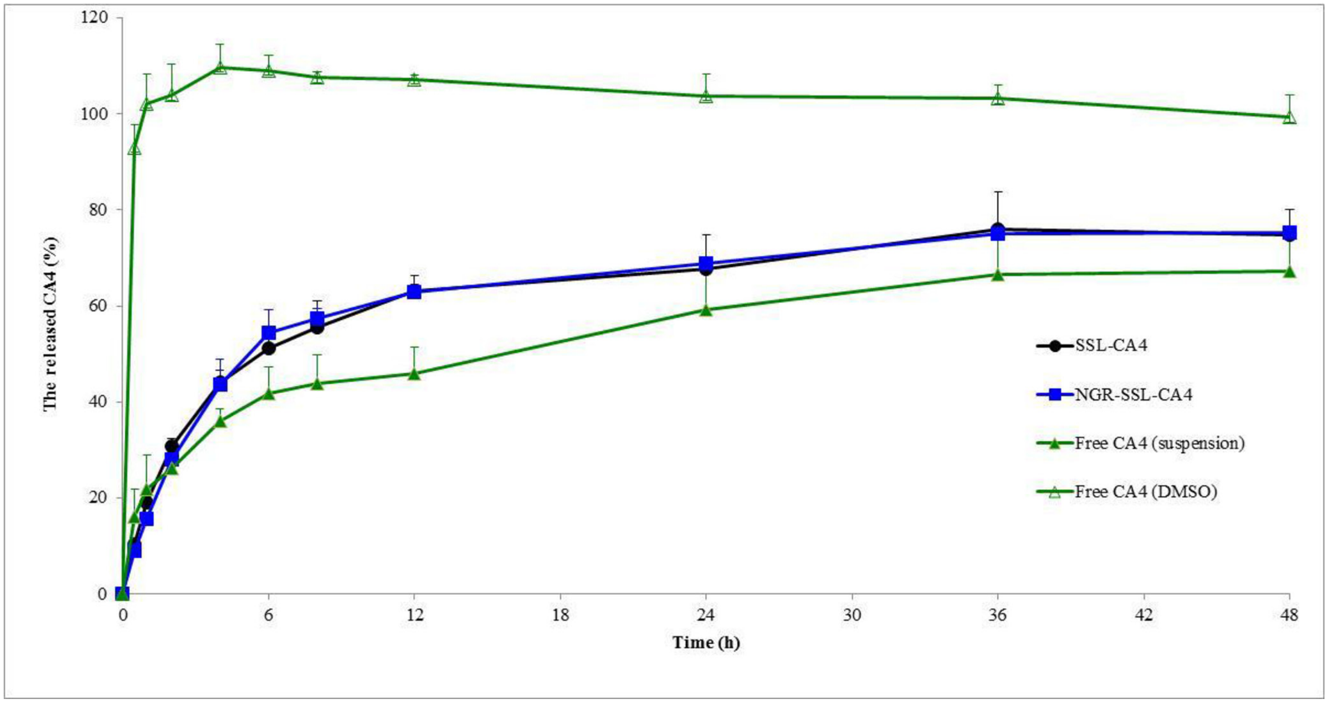

Supplementary Figure S1: The release of CA4 from free CA4 at 37°C in PBS buffer Data represent the mean ± standard deviation (*n* = 3). The free CA4 was selected as CA4 suspended in distilled water or CA4 dissolved in DMSO. The free CA4 (suspension) or free CA4 (DMSO) (0.1 ml, 1 mg/ml) was placed in a dialysis tube, respectively, and then determined according to the "*In vitro* release experiment". The results indicated that the released CA4 from free CA4 (suspension) were lower than that from NGR-SSL-CA4 or SSL-CA4, indicating that the poor solubility of CA4 in distilled water. In contrast, the dissolved CA4 (free CA4 in DMSO) could release completely at 0.5 h, indicating that the dissolved CA4 could fast transport across the dialysis membrane to the released medium.
